# Drawing as a window to emotion with insights from tech-transformed participant images

**DOI:** 10.1038/s41598-024-60532-6

**Published:** 2024-05-21

**Authors:** Hui-Ching Weng, Liang-Yun Huang, Longchar Imcha, Pi-Chun Huang, Cheng-Ta Yang, Chung-Ying Lin, Pin-Hui Li

**Affiliations:** 1 Institute of Allied Health Sciences, College of Medicine, National Cheng Kung University, 1 University Road, East Dist., Tainan, 701401 Taiwan; 2 Institute of Gerontology, College of Medicine, National Cheng Kung University, 1 University Road, East Dist., Tainan, 701401 Taiwan; 3 Institute of International Business, College of Management, National Cheng Kung University, Tainan, Taiwan; 4 Department of Psychology, College of Social Sciences, National Cheng Kung University, Tainan, Taiwan; 5 Graduate Institute of Health and Biotechnology Law, Taipei Medical University, Taipei, Taiwan; 6 Biostatistics Consulting Center, National Cheng Kung University Hospital, College of Medicine, National Cheng Kung University, Tainan, Taiwan; 7 Department of Occupational Therapy, College of Medicine, National Cheng Kung University, Tainan, Taiwan; 8 Department of Public Health, College of Medicine, National Cheng Kung University, Tainan, Taiwan; 9 Department of Engineering Science, College of Engineering, National Cheng Kung University, Tainan, Taiwan

**Keywords:** Psychology, Human behaviour

## Abstract

This study delves into expressing primary emotions anger, happiness, sadness, and fear through drawings. Moving beyond the well-researched color-emotion link, it explores under-examined aspects like spatial concepts and drawing styles. Employing Python and OpenCV for objective analysis, we make a breakthrough by converting subjective perceptions into measurable data through 728 digital images from 182 university students. For the prominent color chosen for each emotion, the majority of participants chose red for anger (73.11%), yellow for happiness (17.8%), blue for sadness (51.1%), and black for fear (40.7%). Happiness led with the highest saturation (68.52%) and brightness (75.44%) percentages, while fear recorded the lowest in both categories (47.33% saturation, 48.78% brightness). Fear, however, topped in color fill percentage (35.49%), with happiness at the lowest (25.14%). Tangible imagery prevailed (71.43–83.52%), with abstract styles peaking in fear representations (28.57%). Facial expressions were a common element (41.76–49.45%). The study achieved an 81.3% predictive accuracy for anger, higher than the 71.3% overall average. Future research can build on these results by improving technological methods to quantify more aspects of drawing content. Investigating a more comprehensive array of emotions and examining factors influencing emotional drawing styles will further our understanding of visual-emotional communication.

## Introduction

The art of drawings is a substantial conduit for delving into the rich complexities of human emotions, facilitating unique insights into non-verbal emotional expressions. With the growth of digital technologies, we are afforded broader opportunities for investigating and understanding these expressions, thereby opening new avenues of research. The progression from emotional expression to perception, culminating in inference, is complex and challenging^1^. I-SKE framework captures the essence of aesthetic experience through the interplay of sensation, knowledge, and emotion, all set in motion by the beholder's intention^2^. Within the domain of sensation, the connection between color and emotion, which acts as an echo of the theory of arousal and valence^3^, has been well-studied^4–12^. Beyond color, our study also explores the concepts of space and drawing styles, relating these elements to theories of arousal and valence, as well as to notions of approach and avoidance. By integrating participant-generated visual methodologies^13^, our study addresses methodological limitations highlighted in previous research. It offers a fresh perspective on the relationships between drawings and emotion, considering the content of emotional expression.

Firstly, previous studies have deepened our insight into the intricate relationship between colors and emotions^4–12^, which mostly echo the theory of arousal and valence^14^. Previous studies typically offer participants a predefined color range for pairing colors with emotions Earlier research often presents participants with a specific range of colors for associating colors with emotions^4–12^. While this method has its merits, it may constrain the genuine expression of emotions.

Secondly, drawing-emotion awareness and recognition hinge on content. Perceptions can differ when the same color appears on different facial expressions or in varying circumstances^15^, underscoring the role of contents and individual factors in color-emotion association studies. Thirdly, there are methodological challenges. While drawings offer a creative and insightful method for capturing emotional expressions, their interpretation and qualitative analysis are inherently subjective, limiting research in this area^16^. Researchers are concentrating on abstract drawings involving color or lines^4^ and stroke techniques^17,18^. However, there appears to be a need for studies specifically examining space or drawing styles and their relation to emotion in the literature. This study employs participant-generated visual methodologies^13^, to bridge the gap in existing literature and enhance authenticity. It treats participants as active 'knowers' rather than passive observers who merely select colors from limited choices.

We aimed to answer the following research questions:

First, did the colors used in drawings vary among the four primary emotions: anger, happiness, sadness, and fear? How were they associated with the theory of arousal and valence?

Second, was the concept of space in drawings related to the theory of approach and avoidance of space?

Third, we sought to classify the depicting styles to determine if they differed by emotions.

Fourth, how did these variables predict the four emotions?

This study involved 182 university students creating drawings to express four primary emotions: anger, happiness, sadness, and fear. Utilizing Python and OpenCV, this study analyzed these digital drawings by converting previously human-perceivable subtleties into quantifiable data. This utilization of digital technology enhances our understanding of non-verbal emotional expressions.

## Literature review

### Drawings and emotion

Previous research suggests that the act of drawings can serve as a medium for the disclosure of emotional expressions^17,19,20^. Drawings encompass a variety of elements such as color, comparison, content, etc. Overall, research analyzing drawings to uncover emotions follows two main directions: The first approach involves quantitative methods focusing on color-pairing. In contrast, the second employs qualitative or mixed methods relying on human judgment.

For decades, the nexus between color and emotion has garnered academic attention. One study highlighted the significant emotional effects of saturation and brightness^11^. Preferred hues included blue, red–purple, and blue-green, whereas yellow was less favored. Another study indicated that primary hues, notably green, triggered positive emotions, which evoked relaxation^8^. Achromatic shades saw white as the frontrunner in positivity. Lastly, a recent study revealed that saturated, bright colors resonated with heightened arousal, with a notable arousal gradient from blue to red^21^.

Research on emotion-color associations primarily occurs in controlled settings. These investigations often use emotional words^5^ or specific colors^9,12,21^ to deduce emotional links. One study established the foundation by identifying a low-to-moderate consensus on color-emotion pairings^4^. Subsequent investigations by Jonauskaite et al.^22^ delved deeper, notably in 2019, linking hues, lightness, and chroma to specific emotions yellow to joy, yellow-green to relaxation, and lighter shades to positivity. This pivotal shift, primarily concerning young adults, underscored age's impact on color-emotion perception. Their landmark 2023 study (n = 7393) spanning 31 countries revealed age-related nuances in color-emotion associations, demonstrating a universal yet age-distinct connection; older adults showed more positive, specific color responses, contrasting with adolescents' less positive bias^7^.

Reynolds et al. measured heart drawings using image analysis software correlated with anxiety and depression^23^. Participants paper drawings were scanned and analyzed based on the area and height. Their findings indicated that larger hearts were associated with increased heart-specific anxiety and negative perceptions of illness among heart failure patients. One study began by employing content analysis on 314 paper drawings, accompanied by explanations from the children or notes by their teachers^24^. The researcher examined symbols and colors and classified them into six fear categories. Results identified prevalent emotions and fears, with animal-related fears being the most common. One recent study trained raters to evaluate 17 formal elements in artwork, including color, composition, and space^20^. The study found significant correlations between the combination of movement, dynamic, and contour in artwork and clients' mental health. Another qualitative study with 132 college students, using graphic elicitation to delve into happiness in leisure activities^25^. The research combined participants' drawings and spoken explanations and found a strong link between happiness and leisure aspects like time, space, and activities. Damiano et al. quantitatively decoded emotional expressions in abstract color and line drawings^4^. They noted that color drawings by non-artists expressed emotions more clearly than those by artists. Previous investigations into emotions through drawing analysis have advanced slowly and sparingly. However, the impact of depiction style, including abstract versus tangible expressions or spatial elements, still needs to be explored. While drawing content analysis often relies on subjective judgments by human raters, computers are generally employed to examine objective attributes such as color, size, and line characteristics.

While the experimental limitations of these studies may restrict their real-world applicability, employing qualitative methods for analyzing drawings allows for a nuanced capture of subjective emotional expressions. Using mixed methods in drawings analysis could offer a more comprehensive understanding of emotional experiences.

### Psychological theoretical framework for emotional expression

Emotions combine physiological and cognitive elements, influencing behavior. Scholars differ on basic emotions. Ekman and Davidson^26^ list happiness, anger, fear, sadness, disgust, surprise, and contempt, while Tomkins^27^ includes interest, enjoyment, surprise, distress, fear, anger, etc. Alternatively, some researchers hierarchically organize emotions, categorizing them as positive or negative and further subdividing them into more specific subcategories introduced the circumplex model of affect, which posits that all affective states are determined by two distinct dimensions: valence and arousal^3,28^. According to this model, emotions can be categorized based on their position along these dimensions. For instance, happiness is associated with positive valence and high arousal; sadness is linked to negative valence and low arousal; anger and fear are typically associated with negative valence and high arousal.

According to Schachter and Singer^14^, the experience and recognition of emotions depend on general physical arousal and the interpretation of that arousal based on environmental cues, emphasizing the cognitive process known as appraisal. Appraisal theories of emotion, well-established in psychology, shed light on individual variations in emotions and how a broad range of emotions arise^29^. Consequently, emotions are not directly triggered by objective features or events in the world but rather through an indirect inference.

The universality of emotions is evident in basic emotional expressions^26^. However, how these emotions are expressed, especially in digital communication, reflects broader cultural shifts. Building on this, Liao^10^ found that the impact of color on the affective interpretation of emoticons significantly enriches our understanding of emotional expression in digital forms, showing that both the affective tone of an emoticon and its background colors can significantly influence its emotional potency in computer-mediated exchanges. Emojis dominate modern communication, especially among Gen Z and Millennials^30^. The research underscores their rapid processing of words^31^ and prominence in virtual workplaces^32^. This shift, influenced by cultural transitions, could reshape emotional expression patterns in digital communication.

### Emerging technologies and the advancement of emotion recognition

Advanced technology is propelling emotion recognition research, simplifying both data collection and the process of emotion identification. These methods are unobtrusive, causing minimal disruption to respondents. Jonauskaite et al. developed a color picker program that allowed 88 participants to select their most and least preferred colors in different contexts, revealing precise color ranges and preferences based on personal experiences^33^. One study evaluated the emotions of 129 subjects through writing and drawing, emphasizing the importance of timing and ductus in strokes^34^. They used Random Forest classifiers for feature analysis, finding depression writing accuracy at 67.8%, drawing accuracy at 71.6%, and a combined accuracy of 71.2%. Another study investigated the attributes of drawing strokes with positional pens technology, suggesting that these strokes could offer insights into a user's cognitive state^18^. One recent study utilized the Emothaw database to detect depression, anxiety, and stress from handwriting and drawing, achieving an average accuracy of 71.06% for depression, 57.93% for anxiety, and 56.93% for stress by employing the leave-one-out technique with a radial basis SVM model^16^. Another recent study analyzed handwriting and drawing in 49 varied mental health status participants^35^. Data were collected using an INTUOS WACOM series of four digital tablets and categorized into pressure, ductus, time, space, and inclination, with stroke counts varying significantly between groups, correlating with depression severity. Damiano et al. utilized computational methods to analyze emotions in drawings, comparing them to averaged reference sets per emotion category and employing histograms of contour features for line drawings^4^. This approach effectively predicted emotions by matching drawings to emotion-specific statistical profiles, demonstrating the technique's accuracy in emotion recognition.

Collectively, these studies underscore the transformative impact of technology on emotion recognition research. From analyzing color-emotion associations to handwriting and drawing, emerging technologies have broadened the horizon for understanding complex emotional states.

## Methods

### Data collection

Initially, we received samples from 202 participants, which were reduced to 182 after removing unreadable or incorrect images. However, 67 participants opted out (i.e., they requested data destruction post-study and refused data sharing for further research). Accordingly, the drawings from 115 participants totaling 460 images and the secondary data from all 182 samples are provided for interested researchers.

All participants were students from elective courses. Additionally, we have explicitly confirmed that all participants were screened for color vision deficiencies, with no such conditions found among the students. Participants received a blank digital JPG file on their smartphones to create drawings depicting four emotions. Participants were instructed to modify the blank JPG using their phone's built-in editing tools or preferred software.

The instruction was: "Draw how you perceive anger, happiness, sadness, and fear. Each emotion should be drawn separately on individual documents; do not combine all emotions into one document. Please ensure that each emotion is depicted on its distinct page. Artistic skills are not essential; simple sketches will do. Please finish within 30 min. We are interested in your expression of emotions in drawing." Participants penned a reflection on their creation, elucidating their rationale. All signed a written "Informed Consent Form," with opt-outs being excluded. While the study included identifiable information, it was exclusively for internal linkage and was anonymized prior to analysis. The research received approval from the Institutional Review Board of National Cheng Kung University Hospital (B-ER-109–424). The relevant guidelines and regulations performed all methods.

### Image processing and measurements

#### Color of image processing and recognition

To analyze image color profiles, we leveraged the strengths of Python software (version 3.10)^36^ and OpenCV (version 4.8.0.74)^37^. Python is a widely used high-level programming language known for its readability and flexibility, ideal for our data analysis needs. OpenCV is a comprehensive library that provides numerous image processing and computer vision algorithms. In our project, we synergistically harnessed these tools: within Python's versatile environment, we utilized OpenCV to process images, convert them into appropriate color spaces, and isolate individual color channels, enabling an accurate quantification of the color information. We first determined the color definitions in the HSV color space using RGB numbers. Previous studies utilized the Munsell color system and other perceptually uniform spaces like Lab, Lch, and xyY, which more accurately reflect human perception^9,12^, emphasizing the significance of employing perceptually uniform color spaces for a more accurate representation of color in digital contexts, as further supported by recent studies^38,39^.

The HSV color space offers a perceptually intuitive color model over RGB, emphasizing hue, saturation, and brightness^40^. Hue (H) varies from 0 to 360 degrees or 0–1, representing color on a wheel, while Saturation (S) and Value (V) quantify intensity and brightness on a 0–100% scale. However, for ease in digital graphics, HSV values are often normalized to 0–255, fitting 8-bit color channels. In Python and OpenCV, Hue is adjusted to 0–180, with S and V maintained at 0–255. This normalization aids in standardizing color representation across software. We detail both the general HSV color system ranges and their specific OpenCV adaptations in Table 1, focusing here exclusively on the OpenCV configuration for clarity.

**Table 1:** Comparative overview of general HSV color system ranges and their adaptations in OpenCV.

Color	HSV color range		OpenCV HSV range	
Range	Lower range	Upper range	Lower range	Upper range
Red	[0, 21, 18]	[20, 100, 100]	[0, 53, 46]	[10, 255, 255]
Red	[292, 21, 18]	[360, 100, 100]	[146, 53, 46]	[180, 255, 255]
Yellow	[18, 21, 22]	[72, 100, 100]	[11, 46, 53]	[36, 255, 255]
Green	[74, 21, 18]	[166, 100, 100]	[37, 46, 53]	[83, 255, 255]
Blue	[168, 21, 18]	[248, 100, 100]	[84, 53, 46]	[124, 255, 255]
Purple	[250, 21, 18]	[290, 100, 100]	[125, 53, 46]	[145, 255, 255]
Gray	[0, 0, 27]	[360, 10, 90]	[0, 0, 71]	[180, 25, 229]
Black	[0, 0, 0]	[360, 100, 27]	[0, 0, 0]	[180, 255, 70]
White	[0, 0, 90]	[360, 11, 100]	[0, 0, 230]	[180, 30, 255]

To analyze color profiles in images, we defined six key variables. The operational definitions for these variables are provided below.

1. Prominent color: this variable identifies the primary color associated with each emotion. For instance, if red is determined to be the prominent color for an emotion, it is coded as "1"; all other colors are coded as "0".
2. Color Percentage: this quantifies the extent to which color fills an image representing a specific emotion, calculated as the percentage of the image's pixels of the specified color versus the total pixels.
3. Number of colors used: seven colors were utilized in the digital canvas for expressive purposes, excluding white, as it represented a blank canvas.
4. Saturation percentage: saturation measures color intensity within the HSV model, ranging from 0 to 100%. To calculate the saturation percentage, the average component from the HSV color space is used^40,41^.
5. Brightness percentage: it represents the perceived intensity or luminance of a color, indicating how much it deviates from a neutral color of the same saturation, such as a gray shade with an equivalent value^42^.
6. Color fill percentage: the proportion of the canvas occupied by color.
7. Image coverage percentage: the proportion of the subject's canvas, including any internal blank spaces.

#### Types of emotional depictions

Since we are interested in the styles participants drew, their images varied widely from realistic to abstract forms, including free-flowing lines. However, drawing inconsistencies makes these styles difficult for programming and learning algorithms to recognize and quantify. Three research assistants categorized content and depiction styles. After refining the categorization criteria over four iterations, the Kappa statistic showed that their agreement ranged between 85 and 92%.

**Figure 1:**
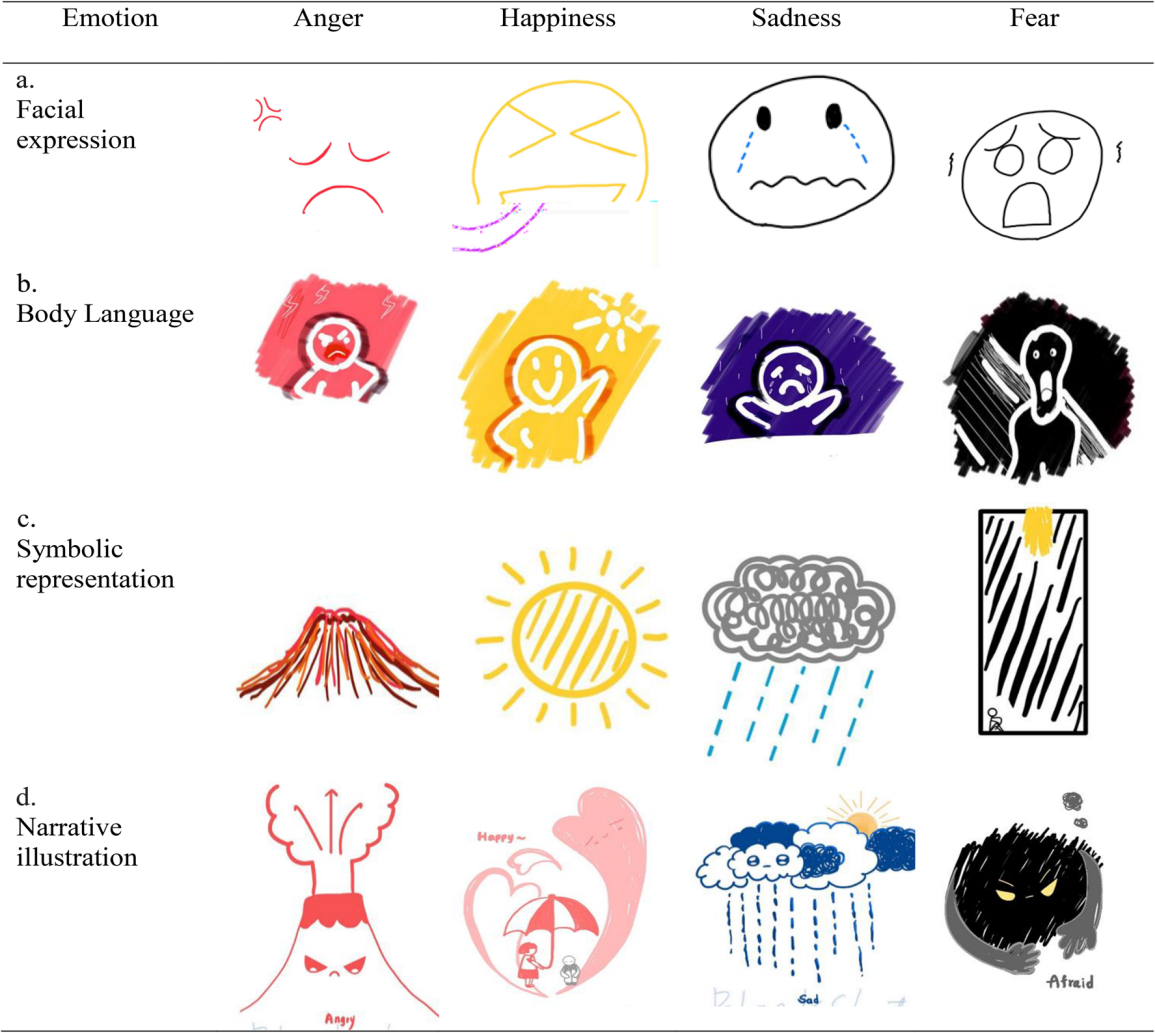
Tangible examples of visual representation across four emotions.

**Figure 2:**
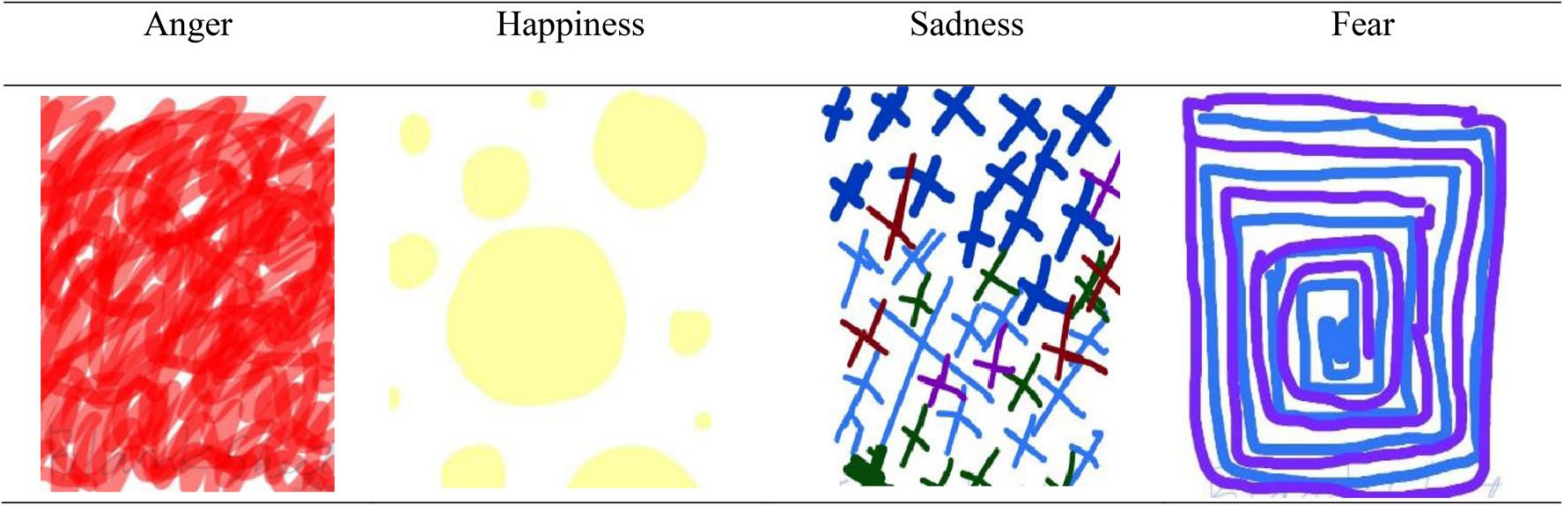
Abstract examples of visual representation across four emotions.

Respondents' drawings representing four emotions were categorized as tangible (Fig. 1) or abstract (Fig. 2). The operational definitions for each are as follows:

*Tangible emotion depictions* (1) Facial expressions: drawings primarily focused on faces expressing emotions. (2) Body language: drawings focused on body postures or movements expres90 sing emotions. (3) Symbolic representation: drawings that only use symbols to represent emotions. (4) Narrative illustration: drawings that tell a story or situation leading to the depicted emotion.

*Abstract emotion depictions* Ambiguous Expressions: drawings where the depiction of emotion is abstract or non-obvious.

#### Data analyses

All analyses were performed using SPSS version 27^43^. Descriptive statistics provided insights into participants' characteristics and highlighted themes in drawings conveying four emotions. We delved deeper using inferential methods. Chi-square and ANOVA tests were utilized to assess differences in expressions of emotion within drawings. Multinomial logistic regression was used to classify emotions in drawings using variables like prominent colors, saturation, brightness, color count, fill, coverage, and five depiction styles (as shown in Figs. 1 and 2). This method employs an iterative maximum-likelihood algorithm for parameter estimation and excels at categorizing subjects using predictor variables^44^. To ensure the reliability and accuracy of our model, we reinforced it using bootstrapping with 5000 resamples, a strategy rooted in statistics^45^. Additionally, we utilized dummy variables for color percentages to avoid the collinearity issue. The model's precision was gauged with an accuracy metric, and a classification table juxtaposed predicted and actual emotional labels, reflecting model efficacy^46^.

## Results

### Comparison of the drawings across different emotions

Table 2 details the demographics of 182 study participants, with an average age of 23.07 (SD = 5.40). Gender distribution includes 106 females (58.2%) and 76 males (41.8%). The majority are undergraduates (86.8%), with fields of study primarily in Medicine (60.4%), followed by Social Science (20.9%), Science (7.1%), Liberal Arts (6.6%), and Engineering (4.9%).

**Table 2:** Demographic information (n = 182).

	n	%
*Sex*		
Male	76	(41.8)
Female	106	(58.2)
*College*		
Engineer	9	(4.9)
Liberal Art	12	(6.6)
Medicine	110	(60.4)
Science	13	(7.1)
Social Science	38	(20.9)
*University students*		
Undergraduate	158	(86.8)
Postgraduate	24	(13.2)

**Figure 3:**
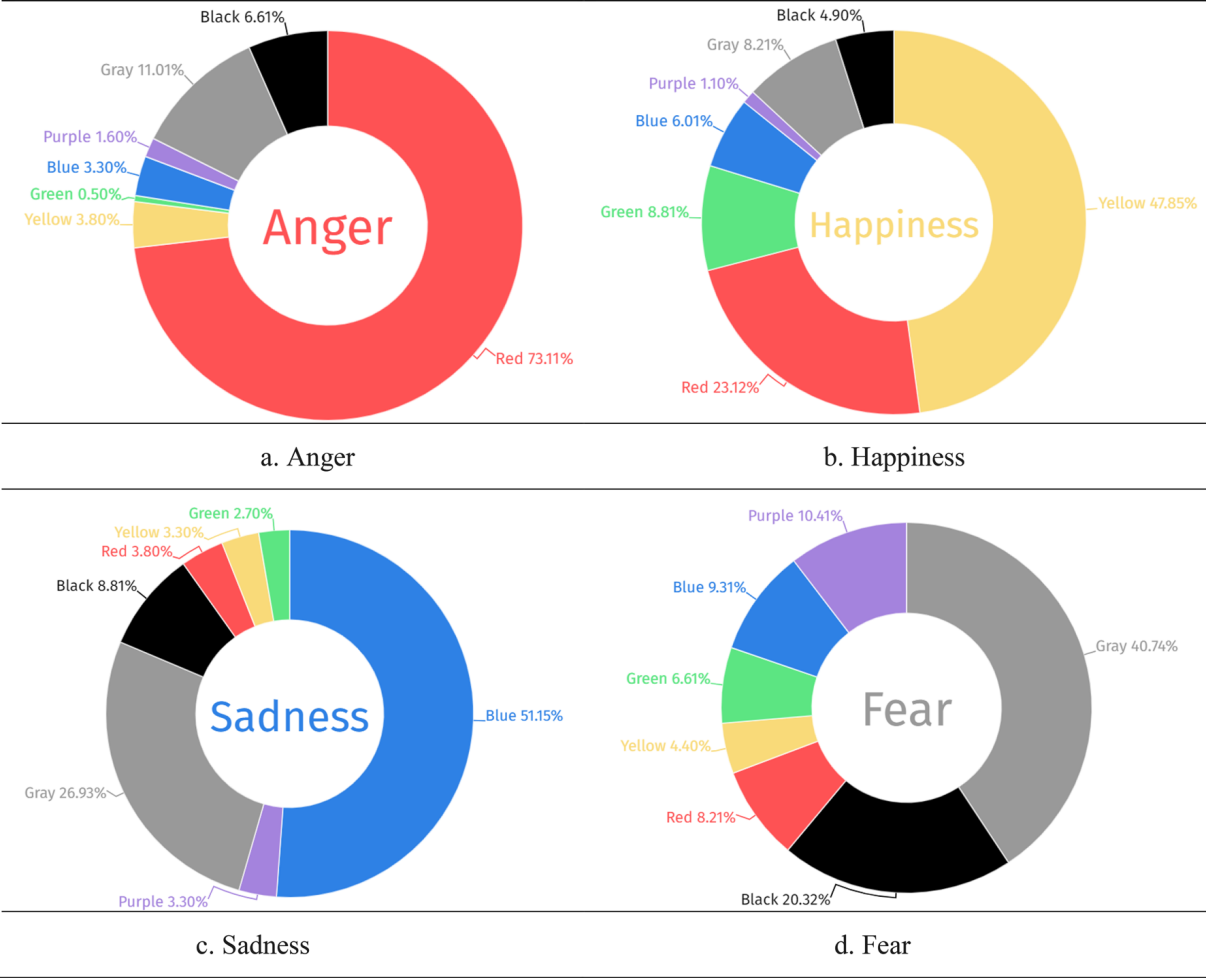
Prominent colors usage by four emotions (n = 182).

Figure 3 presents the prominent color chosen for each emotion, the majority of participants chose red for anger (73.11%), yellow for happiness (47.8%), blue for sadness (51.2%), and black for fear (40.7%). Table 3 displays the significant differences in prominent color choices, color usage, color-filled percentage, brightness, and saturation across four emotions (Chi square = 664.882, *p* < 0.001), with no significant differences in the number of colors used and image coverage. Based on the average usage percentages of colors in images, the most commonly used color for the emotion of anger was red (72.27%). Happiness had yellow (49.9%) as the predominant choice. Sadness was typically depicted using blue (51.66%). Fear's frequently selected colors included black (33.92%). Happiness had the highest saturation percentage at 68.52%, while fear had the lowest at 47.33%. Happiness had the highest percentage of brightness (75.44%), while fear had the lowest brightness (48.78%). Fear had the highest color fill percentage of the four emotions at 35.49%, while happiness recorded the lowest at 25.14%.

**Table 3:** Comparisons of colors used, saturation, and brightness by four emotions (n = 182).

	Anger		Happiness		Sadness		Fear		F	*p*
	Mean	(S.D.)	Mean	(S.D.)	Mean	(S.D.)	Mean	(S.D.)		
*Color percentage*										
Red	72.27	(35.72)	21.92	(34.77)	3.26	(14.86)	8.14	(22.44)	226.339	< 0.001
Yellow	5.1	(15.97)	49.9	(44.21)	4.44	(17)	4.2	(15.72)	136.168	< 0.001
Green	0.58	(5.65)	7.65	(20.92)	3.34	(15.58)	6.76	(22.55)	6.298	< 0.001
Blue	4.21	(17.92)	6.29	(20.89)	51.66	(44.32)	10.49	(26.89)	106.835	< 0.001
Purple	1.59	(10.85)	1.81	(9.6)	3.8	(16.7)	10.06	(27.64)	9.103	< 0.001
Gray	4.64	(17.37)	3.34	(15.69)	17.85	(34.34)	26.43	(39.9)	26.835	< 0.001
Black	11.61	(26.51)	9.09	(25.01)	15.65	(30.58)	33.92	(42.98)	22.349	< 0.001
Color number	1.69	(0.87)	1.81	(1.10)	1.74	(1.00)	1.77	(1.22)	0.439	0.725
Saturation (%)	65.62	(21.46)	68.52	(21.24)	63.94	(25.18)	47.33	(32.46)	25.47	< 0.001
Brightness (%)	60.34	(17.46)	75.44	(14.93)	59.47	(18.21)	48.78	(23.02)	63.009	< 0.001
Color fill (%)	30.73	(18.45)	25.14	(15.3)	26.77	(19.28)	35.49	(23.47)	10.354	< 0.001
Image coverage (%)	87.17	(11.22)	87.23	(9.35)	85.38	(10.47)	85.25	(12.67)	1.777	0.15

*S.D.* Standard deviation.

Table 4 classifies respondents' emotional depictions into two categories across four emotions. Non-abstract styles dominated at 71–84%, while abstract styles peaked for fear (28.57%) and dipped for happiness (16.48%). Significant differences were observed among the four tangible emotion styles (*p* < 0.05 ~ 0.001), except for facial expressions (*p* = 0.47). Tangible categories, such as facial expressions and symbolic representations, were dominant, ranging from 49.5% (anger) to 41.76% (fear) and from 47.8% (anger) to 24.18% (fear), respectively. Anger topped both categories. Fear stood out uniquely among the four types of emotional depictions, attaining the highest rankings in body language (22.53%), narrative illustration (14.29%), and abstract expression (28.57%).

**Table 4:** Types of emotional depictions for four emotions (n = 182).

	Anger		Happiness		Sadness		Fear		F	*p*
	n	%	n	%	n	%	n	%		
*Tangible emotion depictions*										
Facial expressions	90	(49.45)	87	(47.8)	87	(47.8)	76	(41.76)	0.837	0.474
Body language	15	(8.24)	23	(12.64)	25	(13.74)	41	(22.53)	5.414	0.001
Symbolic representation	87	(47.8)	84	(46.15)	56	(30.77)	44	(24.18)	10.89	< 0.001
Narrative illustration	5	(2.75)	13	(7.14)	21	(11.54)	26	(14.29)	5.845	0.001
Abstract emotion depictions	39	(21.43)	30	(16.48)	40	(21.98)	52	(28.57)	2.616	0.05

The overall percentage of prediction rates from multinomial logistic regression was 71.3% (Table 5). Among the emotions, anger had the highest prediction accuracy at 81.3%, followed by sadness at 71.4%, fear at 67.6%, and happiness at 64.8%. Our model exhibits a good fit, with a Pseudo R-Square (Nagelkerke) value of 0.749 and a Chi-Square value of 882.500 (*p* < 0.001).

**Table 5:** Prediction rates for four emotions (n = 182).

Observed	Predicted				
	Anger	Happiness	Sad	Fear	Percent correct (%)
Angry	148	15	3	16	81.3
Happy	36	118	11	17	64.8
Sad	6	10	130	36	71.4
Fearful	13	14	32	123	67.6
Overall percentage	27.9%	21.6%	24.2%	26.4%	71.3

The diagonal values represent correct predictions, while off-diagonal values indicate instances of misprediction.

## Discussion

Our investigation delved into the arena of non-verbal emotional depictions via digital participant drawings. Our findings indicate that color choices in the drawings align closely with those reported in previous studies^4–12^: red was the dominant color for anger (73.1%), yellow for happiness (47.8%), blue for sadness (51.1%), and black for fear (40.7%). Fear had the most color fill at 35.95%, and fear had the smallest image coverage at 84.63%. Non-abstract styles dominated at 74–85%, while abstract styles peaked for fear (28.5%) and dipped for happiness (16.5%). The model predicts emotions with 71.3% average accuracy, excelling in anger prediction at 81.3%

### Bridging valence-arousal and approach-avoidance theories

As elaborated, the theoretical constructs of valence and arousal offer a nuanced lens for dissecting the intricacies of emotional experience^3,47^. These constructs categorize emotions based on their hedonic tone and level of physiological arousal, thereby providing a foundational scaffold upon which our empirical findings can be mapped and interpreted. Our study reflects this model: red (73.1% in anger drawings) aligns with high arousal and negative valence, indicative of anger intensity. Yellow (47.8% in happiness drawings) varies in arousal but is positively valenced, signifying joy. Blue (51.1% in sadness drawings) shows low arousal and negative valence, depicting melancholy. Black (40.7% in fear drawings) suggests moderate arousal and negative valence, akin to fear's uncertainty and anxiety.

Our results also resonate with previous studies^12,21^, who linked saturated colors to higher arousal. In our study, happiness exhibited the highest saturation at 68.52%, contrasting with fear's lower saturation (47.33%), which paradoxically hinted at heightened arousal and avoidance behavior typically associated with fear. Moreover, the presence of 75.44% high brightness in drawings associated with happiness indicates a positive valence. This observation is supported by Lin et al.^48^, who demonstrated that the perception of colored photos changes with valence and saturation. Thus, our study underscores the complex role of color attributes in emotional representation and affective judgment, providing practical insights into the connection between color use in drawings and emotional states as defined by arousal and valence.

Our study observed intriguing contrasts in color fill percentages associated with different emotions. 'Fear' occupied 35.49% of the color fill, possibly as a therapeutic outlet for emotional relief^49^. In contrast, 'Happiness' represented only 25.14%, indicating a lesser need for expression. Our findings, interpreted through the approach-avoidance theory, reveal nuanced emotional narratives. Avoidance motivations, reflected in 'Fear's' vivid coloration, heighten alertness. In contrast, 'Happiness,' associated with approach motivations, appears more subdued, subtly inviting positive experiences that align with theories where color variations embody distinct emotional motivations.

### Depiction styles: tangible vs. abstract

Our data exhibits a clear preference for tangible emotional illustration styles. Extensive evidence supports the universality of facial expressions as indicators of emotional states^50,51^. Our study corroborated this, revealing that many participants opted for tangible representations, predominantly through facial expressions and symbols. The prevalence of anger, attributed to its intensity and explicit nature, dominates the tangible emotional depictions. Our findings echo one study that emphasized the pivotal role of facial expressions in emotion conveyance, noting that masks reduce recognition by 31%, except for anger^51^. Narrative illustrations were seldom utilized across emotions, with fear at the highest (14.29%), which might be due to their complexity and time demands. They require advanced artistic skills and intricate narrative structures. Fear's relatively higher use implies its compatibility with narratives, aligning with its suspenseful nature. Our study reveals that fear comprises the largest percentage (28.57%) of abstract depictions in our artwork, a finding consistent with the vigilance-avoidance hypothesis^52^. This hypothesis offers a nuanced perspective on fear. It highlights its prevalence in abstract expressions and suggests that socially anxious individuals may initially be drawn to fear stimuli before eventually avoiding them, potentially internalizing the emotion. Furthermore, one recent study^53^ link uncertainty to negative emotions like fear and anxiety, implying that the abstract nature of fear in our artwork conveys emotional complexity and uncertainty. Again, this aligns with the perspective that fear often manifests internally and is less overt, further emphasizing its intricate relationship with uncertainty^54^.

### Communication style

As social media continues to reshape our digital landscape, the articulation of emotions mirrors profound cultural transformations. In the tangible categories of emotional portrayal, facial expressions ranged from 49.45% (anger) to 41.76% (fear), as illustrated in Table 4. Our study, involving predominantly college students, aligns with current digital communication trends. The Adobe 2022 U.S. Emoji Trend Report emphasizes the extensive use of emojis, particularly among Gen Z and Millennials^30^. Facial or emoji stimuli process more rapidly than words, highlighting the efficiency of visual symbols in emotional communication^31^. Another recent study indicates that emojis elicit high arousal and that discrete emotions are most recognizable in emojis, followed by human faces and emoticons^55^. An overwhelming 91% of respondents use emojis to lighten conversations, and 69% prefer expressing emotions through emojis over text-only conversations^30^. Notably, post-COVID-19, more people have become accustomed to virtual workplaces, with collaborators increasingly using non-textual responses to cultivate interpersonal bonds in informal settings^32^. This shift influenced how respondents visually expressed emotions, mirroring the emojis or stickers they frequently used. However, despite these correlations, significant limitations must be acknowledged. Emotional expression through simplified facial representations, akin to emojis, might restrain creative expression, potentially diminishing the complexity of emotional communication. While these methods enable quick communication, they may need more depth, potentially failing to serve more nuanced forms of emotional expression adequately. Moreover, ubiquitous representations may homogenize emotional expression, compromising individual uniqueness due to their inherent socialization.

### Prediction

In our study, we implemented multinomial logistic regression to classify emotions, achieving an average prediction accuracy of 71.3% across all emotions, with a notable 81.3% accuracy in predicting anger. Our model uniquely incorporated various factors, including saturation, brightness, image coverage, color fill, and depiction style, distinguishing it from traditional studies focusing mainly on color-emotion associations. Comparatively, one study assessed emotions through writing and drawing, utilizing Random Forest classifiers to report a 71.6% accuracy in detecting depression from drawings^34^. Similarly, Nolazco-Flores et al. used handwriting and drawing analyses from the Emothaw database, employing a radial basis SVM model to achieve varying accuracies for depression, anxiety, and stress^16^. Another study^35^ investigated stroke count variations in handwriting and drawing tasks among different depression severity groups. Distinctively, our study's drawings were participant-generated, allowing a broader spectrum of emotional expression through free drawing as opposed to the specific objects like trees or houses often used in other studies. This approach resulted in a richer variety of indicators for emotion prediction. Future research can enhance emotion prediction techniques by building on our methodology of using diverse, participant-generated drawings for more comprehensive nonverbal analyses.

### Implications

First, our drawing-based approach, akin to art psychotherapy, offers a transformative and empathetic method for emotion exploration, avoiding the invasiveness of traditional questionnaires. It creates a relaxing environment, circumventing the discomfort of direct questioning. These insights demonstrate the potential of drawing as an alternative method for detecting and engaging with participants' mental challenges and thought processes, and this approach holds promise for application in both practice and education. Second, leveraging Python and OpenCV, we transformed qualitative images into quantitative data, offering a more in-depth analysis of emotional representations. Our findings show an 81.3% accuracy in predicting anger, significantly higher than the 71.3% average for other emotions, indicating more intricate emotion-color perceptions. Thirdly, our study breaks new ground in color-emotion research by using images created by participants, shifting from the traditional approach of predefined color-emotion pairings. This method recognizes respondents as active contributors, enriching our understanding of how viewers interpret and express emotions through imagery.

### Research limitations and future directions

First, the homogeneity of our participants, all university students, narrows our findings' scope and applicability to a broader audience. However, this homogeneity also strengthens, offering a focused exploration of this demographic's emotional and color perceptions. It enables a detailed analysis of patterns, like the gender-specific use of yellow in expressing sadness, providing valuable insights into the emotional nuances of young adults in academia. Secondly, the diverse and intricate drawing styles, especially free-style representations, posed challenges, leading us to lean more on human judgments. Programming improvements might consider hybrid models that combine machine learning with heuristic or semantic analyses. Additional strategies worth exploring include feedback loops, deeper integration of advanced learning algorithms, and enriching the training data pool. Thirdly, the majority of participants preferred tangible representations for expressing emotions. Influences such as drawing habits, recent emotional states, artistic backgrounds, or personality traits could have played a significant role in their choices. Future research should focus on exploring these potential influencing factors.

## Conclusions

In conclusion, our study marks a notable advancement in image-emotion analysis using Python and OpenCV, converting subtle human perceptions into measurable data. The use of drawings as a non-invasive method has proven valuable in both research and practical applications, highlighting its relevance in psychological assessment and educational practices.

## Data Availability

This data has been coded and permanently de-identified. The datasets generated and/or analyzed during the current study are available in the OSF repository at. https://osf.io/4wdgv/?view_only=99ebdb13e1d54b94be3094d64add136d.
